# Highly Efficient and Selective Biosensing of Malathion Utilizing Bioengineered Nanoplatform Based on Fe_3_O_4_

**DOI:** 10.1021/acs.langmuir.5c05560

**Published:** 2026-01-07

**Authors:** Kshitij RB Singh, Pooja Singh, Shyam S. Pandey

**Affiliations:** † Graduate School of Life Science and Systems Engineering, 12924 Kyushu Institute of Technology, 2-4 Hibikino, Wakamatsu, Kitakyushu (808-0196), Japan; ‡ Department of Biotechnology, Indira Gandhi National Tribal University, Amarkantak, Madhya Pradesh (484886), India

## Abstract

Nanobioengineered platforms have emerged as promising candidates for biosensing due to their exceptional surface properties accompanied by enhanced functionalization potential. In this research, biogenically synthesized Fe_3_O_4_ nanoparticles (NPs) have been utilized to fabricate efficient biosensors for the selective and sensitive detection of malathion pesticides. The structural and physicochemical properties of the Fe_3_O_4_ NPs were thoroughly validated, ascertaining their stability and suitability for malathion biosensing. The Fe_3_O_4_ NPs were electrodeposited onto the carbon working electrode of the screen-printed electrode (SPE) via electrophoretic deposition, followed by the immobilization of choline oxidase (ChO) to create the ChO-Fe_3_O_4_ NPs/SPE nanobioengineered electrodes. This biosensor exhibited remarkable sensitivity and specificity toward malathion, with its performance being governed by diffusion layer dynamics, allowing for the reliable detection of malathion across a broad concentration range. With a detection limit of 1.79 μM and a sensitivity of 98.3 μA μM^–1^cm^–2^ over a wide linear range of 1–200 μM, the sensor exhibits high analytical performance that is competitive with previously reported malathion sensors. The sensor exhibited excellent selectivity, precision, and long-term stability, demonstrating its suitability for the rapid and accurate quantification of malathion in agricultural and environmental samples. Furthermore, real-sample analysis using spiked soil samples showed high recovery rates (93.9% to 98.4%), confirming the sensor’s reliability for practical applications. This eco-friendly and cost-effective approach highlights the potential of biogenic nanomaterials in the development of advanced pesticide sensors, thereby contributing to safer environmental practices and enhanced food quality monitoring.

## Graphical abstract

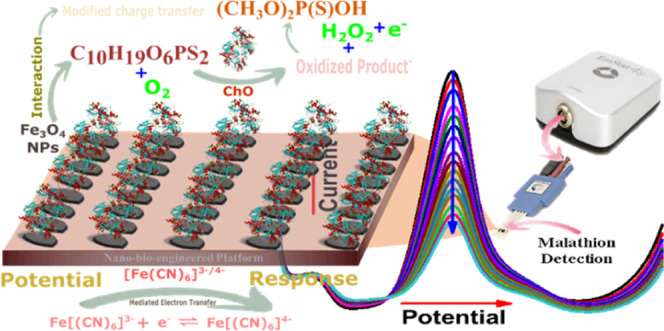

## Introduction

Nanobioengineered platforms have emerged as a transformative approach by leveraging nanotechnology’s potential to integrate nanomaterials with bioreceptors such as enzymes, antibodies, and nucleic acids. This integration enhances the performance of biosensors by providing high sensitivity, selectivity, and efficient interaction with analytes at the nanoscale.^1,2^ Advances in biosensing through nanobiosensor platforms demonstrate the convergence of nanotechnology and biotechnology, enabling rapid and highly sensitive detection platforms applicable in medical diagnostics, environmental monitoring, and food safety. Recent literature also highlights progress in electrochemical biosensing platforms employing magnetic and transition-metal nanomaterials, such as l-cysteine-capped Fe_3_O_4_ NPs embedded in chitosan-MWCNT matrices for pesticide detection,^3^ copper-anchored magnetite nanocomposites for nitrate sensing,^4^ allylamine-capped copper nanoparticle platforms for soil nitrate analysis,^5^ and Ni–NiO-based aptasensors for patulin detection.^6^ These studies emphasize the growing importance of nanostructured interfaces for electrochemical biosensing. Nanobio interfaces play a crucial role in these platforms, acting as both a reaction surface and a barrier between phases. These interfaces rely on colloidal forces and dynamic biophysicochemical interactions, including particle wrapping, protein coronas, and intracellular absorption, all of which enhance the functionality and biocompatibility of biosensors.^7,8^ However, the need to develop biosensors for critical contaminants, such as pesticides, remains paramount, particularly with designs suitable for field applications.^9^

Among the various applications of nanobiosensors, detecting environmental contaminants such as pesticides is critically important. Organophosphates, such as malathion, are widely employed in agriculture to protect crops, including rice, potatoes, and corn, due to their effectiveness against insects and pests. However, their overuse poses severe risks to human health and the environment, including acute toxicity and ecological imbalance.^10^ Traditional methods for detecting malathion, such as gas chromatography and high-performance liquid chromatography, are precise but suffer from drawbacks like high cost, complexity, and lack of portability.^11,12^ Recent advances in biosensors, particularly those using enzyme-modified nanomaterial electrodes, have shown significant promise for overcoming these limitations. Enzyme immobilization on chemically modified nanomaterials offers several advantages, such as increased surface area, enhanced electron transfer kinetics, and optimized enzyme bioactivity, resulting in highly sensitive and selective analyte detection.^13,14^

Fe_3_O_4_ nanoparticles (NPs) synthesized using plant-based green chemistry approaches, such as the use of *Argyreia nervosa* (AN) leaf extract, offer a sustainable route for NP synthesis. AN leaves are rich in bioactive compounds, including flavonoids, alkaloids, saponins, and phenolic acids, which act as natural reducing and stabilizing agents, circumventing the need for hazardous chemicals. This method not only minimizes environmental impact but also yields Fe_3_O_4_ NPs with excellent electrochemical properties, biocompatibility, and stability, making them ideal for biosensor development.^15,16^ In contrast to conventional coprecipitation or sol–gel synthesis, the AN-mediated route minimizes surfactant residues and promotes biofunctional surface formation, which enhances enzyme affinity, electrochemical stability, and overall environmental compatibility.^17^ AN was chosen over other plants due to its high content of bioactive compounds, ease of availability, and historical use in traditional medicine, which highlights its potential for sustainable and efficient NP synthesis. These Fe_3_O_4_ NPs, characterized for their structural and morphological attributes, provide a robust and eco-friendly platform for enzyme immobilization, crucial for developing sensitive and selective biosensors for pesticide detection. Although enzyme-based biosensors for organophosphate pesticides have been extensively reported, most of them depend on acetylcholinesterase (AChE) inhibition mechanisms, which suffer from regeneration issues and poor long-term stability.^18^ In contrast, the present work introduces a noninhibitory, oxidation-based detection pathway employing Choline Oxidase (ChO), in which the sensing signal is proposed to arise from oxidation processes involving ChO, the Fe_3_O_4_ nanofilm, and the redox mediator, leading to electrochemically detectable hydrogen peroxide. This approach avoids inhibition recovery steps, improving the reproducibility, operational stability, and sensor reusability. Furthermore, the biogenically synthesized Fe_3_O_4_ NPs using AN extract provide a previously unexplored green synthesis route, where the plant’s rich polyphenolic and alkaloid content serves as natural reducing and capping agents.^17^ The resulting surface-functionalized Fe_3_O_4_ offers enhanced enzyme affinity, increased conductivity, and superior electron transfer properties. Unlike conventional drop-casting, the use of electrophoretic deposition produces a uniform, adherent, and stable Fe_3_O_4_ nanofilm, ensuring a high enzyme loading and robust electrochemical performance. This integrated strategy of green Fe_3_O_4_ synthesis, electrophoretic deposition-based assembly, and ChO oxidation mechanism provides a unique, ecocompatible nanobioengineered interface for malathion sensing not previously reported in the literature.

Thus, this study introduces (Figure 1) a nanobioengineered platform based on Fe_3_O_4_ NPs synthesized via a green synthesis approach using AN leaf extract. The platform was fabricated on screen-printed electrodes (SPEs) with an immobilized ChO enzyme to form ChO-Fe_3_O_4_ NPs/SPE electrodes. The resulting platform was evaluated for its ability to detect malathion, leveraging the nanobio interface for highly sensitive and selective pesticide sensing in complex matrices. Electrochemical investigations were conducted using a three-electrode SPE system, where interfacial and enzyme kinetics played a critical role in achieving rapid and accurate detection. By establishing an optimal nanobioengineered interface, this study demonstrates the potential of Fe_3_O_4_ NP-based biosensors in addressing the pressing need for sustainable, cost-effective, and field-deployable pesticide detection systems. Additionally, this work opens pathways for integrating biosensors into compact devices for real-time monitoring, offering scalable solutions for food safety, environmental health, and human well-being.

**Figure 1:**
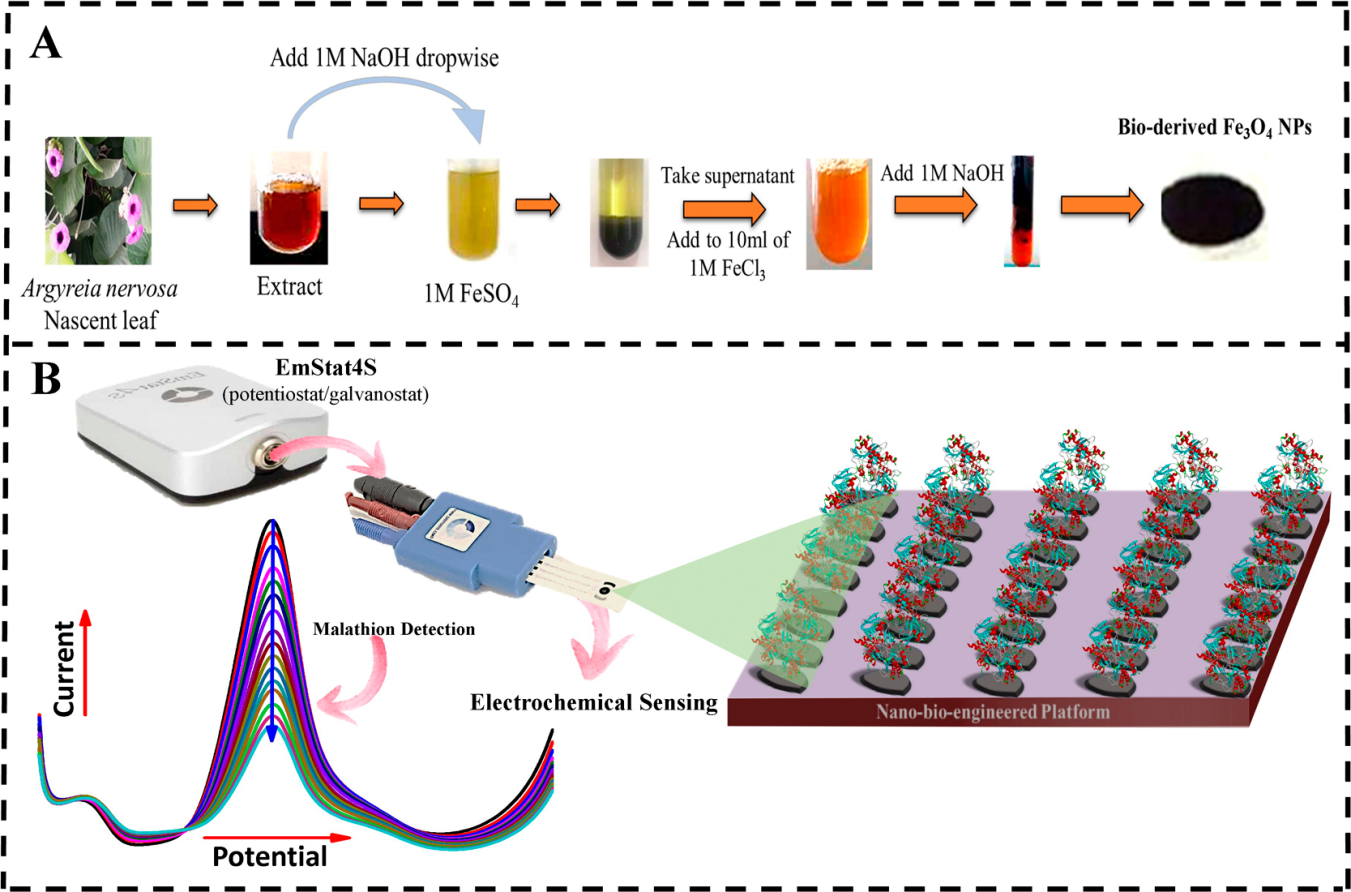
From synthesis to biosensing: (A) Synthesis of Fe_3_O_4_ NPs and (B) nanobioengineered platform fabrication and malathion detection.

## Materials and Methods

The materials and reagents utilized in the study were of analytical grade, and the details are provided in Section S1 of the Supporting Information. Additionally, Section S2 of the Supporting Information includes a list of the equipment used. Also, the optimization of Fe_3_O_4_ NP loading and ChO enzyme concentration was conducted prior to biosensor fabrication (see Supporting Information S7), ensuring maximum catalytic response and reproducibility.

### Fe_3_O_4_ Synthesis and Nanobioengineered Platform Fabrication

AN leaves were processed as per our earlier publication.^13^ Leaves were chopped, ground into a paste, and diluted in Milli-Q water (1:10). The mixture was heated at ∼70 °C for 30 min, cooled, filtered, and stored at 4 °C. For green synthesis (Figure 1A), 10 mL of plant extract was mixed with 1 M ferrous sulfate heptahydrate (FeSO_4_) (1:1 V/V) forming a dark-brown solution. A dropwise 1 M NaOH solution was adjusted to pH ∼12 for precipitation. The addition of Ferric chloride (FeCl_3_) and NaOH leads to the formation of black magnetic NPs, which settled upon exposure to a magnet.

The Fe^2+^ and Fe^3+^ ions required for NP formation originate from FeSO_4_·7H_2_O and FeCl_3_, respectively, which dissociate in the solution. In this synthesis, phytochemicals in the AN leaf extract act as natural reducing and stabilizing agents, facilitating the conversion of Fe^2+^ and Fe^3+^ ions into Fe_3_O_4_ nuclei in an alkaline medium (eq 1). Biomolecules such as flavonoids, tannins, and polyphenols donate electrons, reducing Fe^3+^ to Fe^2+^, while hydroxide ions from NaOH induce coprecipitation. NaOH maintains pH ∼12, aiding hydroxide ion-driven coprecipitation while stabilizing agents in the extract prevent agglomeration. The dehydration and rearrangement of the precipitate lead to the formation of crystalline Fe_3_O_4_ NPs. Finally, the synthesized NPs were washed 4 times with Milli-Q water and centrifuged at 10000 rpm for 10 min each time. The resulting pellets were air-dried in a hot-air oven at 70 °C for 18 h and stored in an airtight container for further characterization and applications.

1 $$2FeSO_{4}.7H_{2}O+Fe{Cl}_{3}+Leaf\;Extract+8NaOH\to {Fe}_{3}O_{4}+2{Na}_{2}SO_{4}+NaCl+Oxidized\;Extract+18H_{2}O$$

Further, to fabricate an Fe_3_O_4_-based nanoplatform, a thin film was fabricated evenly on SPE on the working electrode (carbon [graphite]) in the 0.07 cm^2^ area via electrophoretic deposition (30 V, 20 s). After this, the ChO enzyme was immobilized by drop-casting of a 10 μL enzyme solution and allowing it to rest at room temperature for 24 h in a clean, dry chamber for physical immobilization, forming a ChO-Fe_3_O_4_ NP/SPE nanobioengineered platform. The immobilization used here is simple physisorption; no cross-linker or covalent chemistry was employed. Retention of ChO on the Fe_3_O_4_ film arises from electrostatic interactions and hydrogen bonding between enzyme side chains (–NH/–COO^–^/–OH) and the hydroxylated Fe_3_O_4_ surface, while the EPD nanofilm provides a high area and mixed-valence (Fe^2+^/Fe^3+^) conductivity that supports fast electron transfer during sensing. After enzyme immobilization, the nanobioengineered electrode was rinsed to remove unbound enzymes, stored at 4 °C, and used for biosensing experiments.

## Result and Discussion

### Characterization of Fe_3_O_4_ NPs

Initially, the UV–visible absorption spectrum was recorded for biologically synthesized Fe_3_O_4_ NPs, as shown in Figure 2A. The absorption peak at 252 nm for Fe_3_O_4_ NPs corresponds to a 2.72 eV bandgap (Figure 2B, calculated using eq 2). This indirect optical bandgap of the Fe_3_O_4_ NPs was estimated using a Tauc plot by fitting the linear region of the (α*h*ν)^1/2^ vs *h*ν curve in the range 4.5–4.8 eV and extrapolating to (α*h*ν)^1/2^ = 0. The corresponding intercept yields an apparent indirect transition energy. This bandgap tuning, influenced by particle size and synthesis conditions, enhances the optical properties, confirming their potential in photocatalysis and biosensing applications. These results are consistent with the previous work by Khatun et al.^19^ The indirect energy bandgap was determined using a Tauc plot (eq 2), based on the electronic absorption spectral data. In eq 2, “α” represents the absorption coefficient, and “*h*ν” denotes the photon energy. The parameters “*A*” and “*E*_g_” in the equation correspond to absorbance and the indirect bandgap energy, respectively. Although UV–vis analysis is not directly part of electrochemical detection, the observed bandgap narrowing reflects improved conductivity and electron mobility, which are relevant to biosensor performance.

2 $${\left(\alpha h\nu \right)}^{1/2}=A\left(h\nu -E_{g}\right)$$

**Figure 2:**
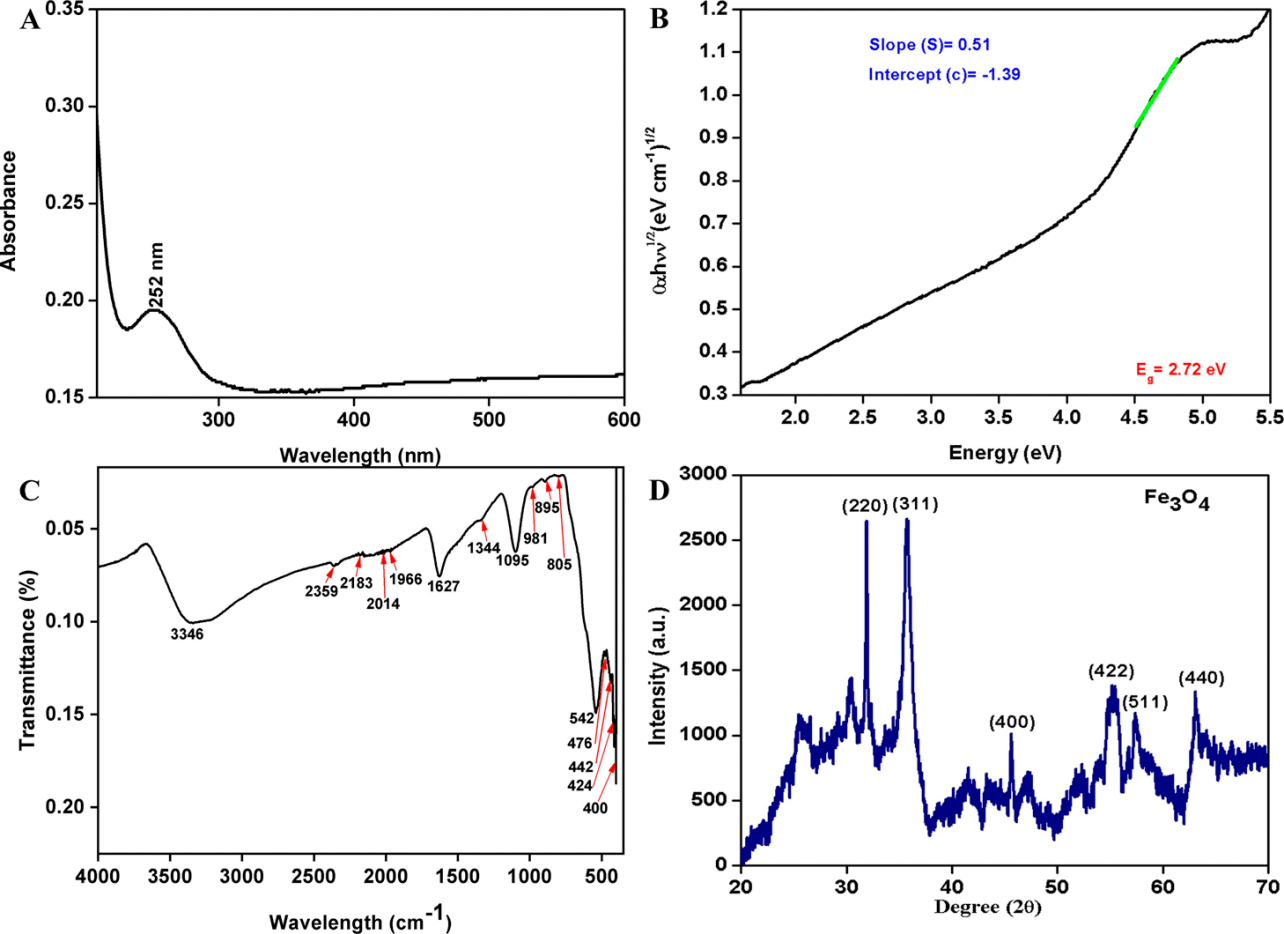
Fe_3_ *O*_4_ NPs: (A) UV–visible, (B) Tauc plot, (C) FTIR spectra, and (D) XRD.

The Fourier transform infrared spectroscopic analysis of Fe_3_O_4_ NPs (Figure 2C) synthesized using AN indicates successful biogenic synthesis through plant-mediated reduction and stabilization. The broad peak near 3400 cm^–1^ corresponds to the O–H stretching of phenolic compounds, which serve as reducing agents during synthesis.^20^ The peak at 1630 cm^–1^ indicates the C=O stretching of carboxylic acids, acting as capping agents.^21^ The peak around 1400 cm^–1^ is attributed to aromatic amine (N–H) groups, while the peak at 1120 cm^–1^ corresponds to C–O stretching from phenolic compounds.^22^ Peaks between 600 and 570 cm^–1^ confirm Fe–O vibrations, validating the formation of Fe_3_O_4_ NPs. Additional peaks near 1200–1100 cm^–1^ could arise from C–O stretching, while minor peaks in the 1000–900 cm^–1^ region suggest contributions from aromatic polyphenols or other plant-derived metabolites, further confirming the role of phytochemicals in NP stabilization. Also, to verify enzyme immobilization on the device, solid-state FTIR was recorded directly from bare SPE, Fe_3_O_4_ NPs/SPE, and ChO-Fe_3_O_4_ NPs/SPE films (Supporting Information S4; Figure S1). The Fe_3_O_4_-coated electrode shows the characteristic Fe–O lattice band at ∼560–580 cm^–1^, confirming the presence of magnetite on SPE. After ChO deposition, new protein bands appear: amide I (C=O stretch, ∼1668 cm^–1^), amide II (N–H bend/C–N stretch, ∼1558 cm^–1^), and amide III (1238–1317 cm^–1^),^23^ together with intensified aliphatic C–H stretches (2923–2852 cm^–1^) and a broadened N–H/O–H envelope (3200–3550 cm^–1^). These features are absent or much weaker on SPE and Fe_3_O_4_ NPs/SPE, and they confirm successful (noncovalent) physisorption of ChO on the Fe_3_O_4_ film, while the Fe–O band remains, indicating the underlying nanofilm is intact.

The X-ray diffraction analysis (XRD) of Fe_3_O_4_ NPs demonstrates well-defined peaks at 2θ values of approximately 31.6°, 35.7°, 45.6°, 55.2°, 57.3°, and 63.1°, corresponding to the (220), (311), (400), (422), (511), and (440) planes, respectively (Figure 2D). These reflections match the standard pattern for Fe_3_O_4_ with an inverse spinel structure [International Center for Diffraction Data (ICDD) reference code: 01-075-0449] with Fe^3+^ and Fe^2+^ ions occupying tetrahedral and octahedral sites. The sharpness of the peaks indicates high crystallinity, and the calculated average crystallite size was found to be 10.36 nm (Table S1 in Supporting Information S3) as determined using Scherrer’s equation (eq 3). In eq 3, “*D*” represents the crystallite size, “λ” is the X-ray wavelength with a value of 1.5406 Å, “*k*” denotes the form factor, “β” refers to the full width at half-maximum of the diffraction peaks, and “θ” corresponds to the Bragg diffraction angle. The XRD analysis confirms the successful synthesis of Fe_3_O_4_ NPs with excellent structural integrity and these findings are consistent with previous literature.^24^

3 $$D=\frac{k\lambda }{\beta \cos\theta }$$

The SEM micrographs (Figure 3A) reveal the morphology of Fe_3_O_4_ NPs, showing aggregated, nearly spherical particles with nanoscale dimensions, confirming structural uniformity. Energy Dispersive X-ray (EDX) analysis (Figure 3B) shows Fe (44.5 at. %, 73.7 wt %) and O (55.5 at. %, 26.3 wt %) are present in stoichiometric ratios for Fe_3_O_4_, validating the elemental composition. Further, elemental mapping (Figure 3C) demonstrates a homogeneous distribution of Fe and O.

**Figure 3:**
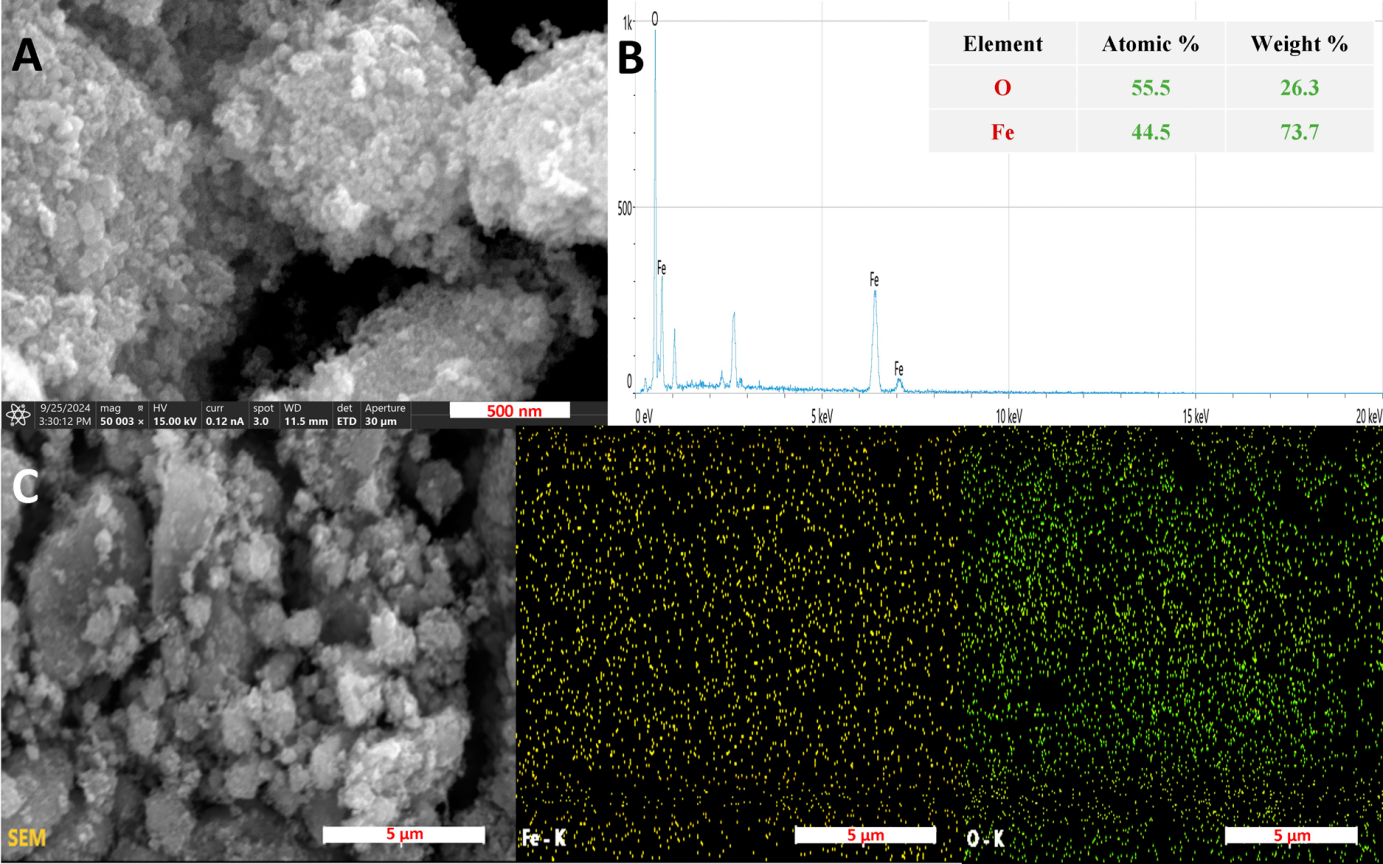
Fe_3_ *O*_4_ NPs: (A) SEM, (B) elemental mapping, and (C) EDX analysis.

The TEM analysis (Figure 4A) of Fe_3_O_4_ NPs reveals their nanoscale size and crystalline nature. The particles are nearly spherical with an average size of 14.25 nm, as indicated by the particle size histogram (inset, Figure 4B). The high-resolution image shows lattice fringes (Figure 4B) with interplanar spacings of 0.22 and 0.18 nm, corresponding to the (311) and (422) planes of XRD, respectively, confirming the inverse spinel structure. The close agreement between crystallite size (10 nm) and particle size (14 nm) indicates minimal aggregation, suggesting that each nanoparticle behaves as a single crystalline domain. These findings align with SEM results, which show aggregated spherical particles and nanoscale morphology. The particle size measured by TEM complements the crystallite size derived from XRD.

**Figure 4:**
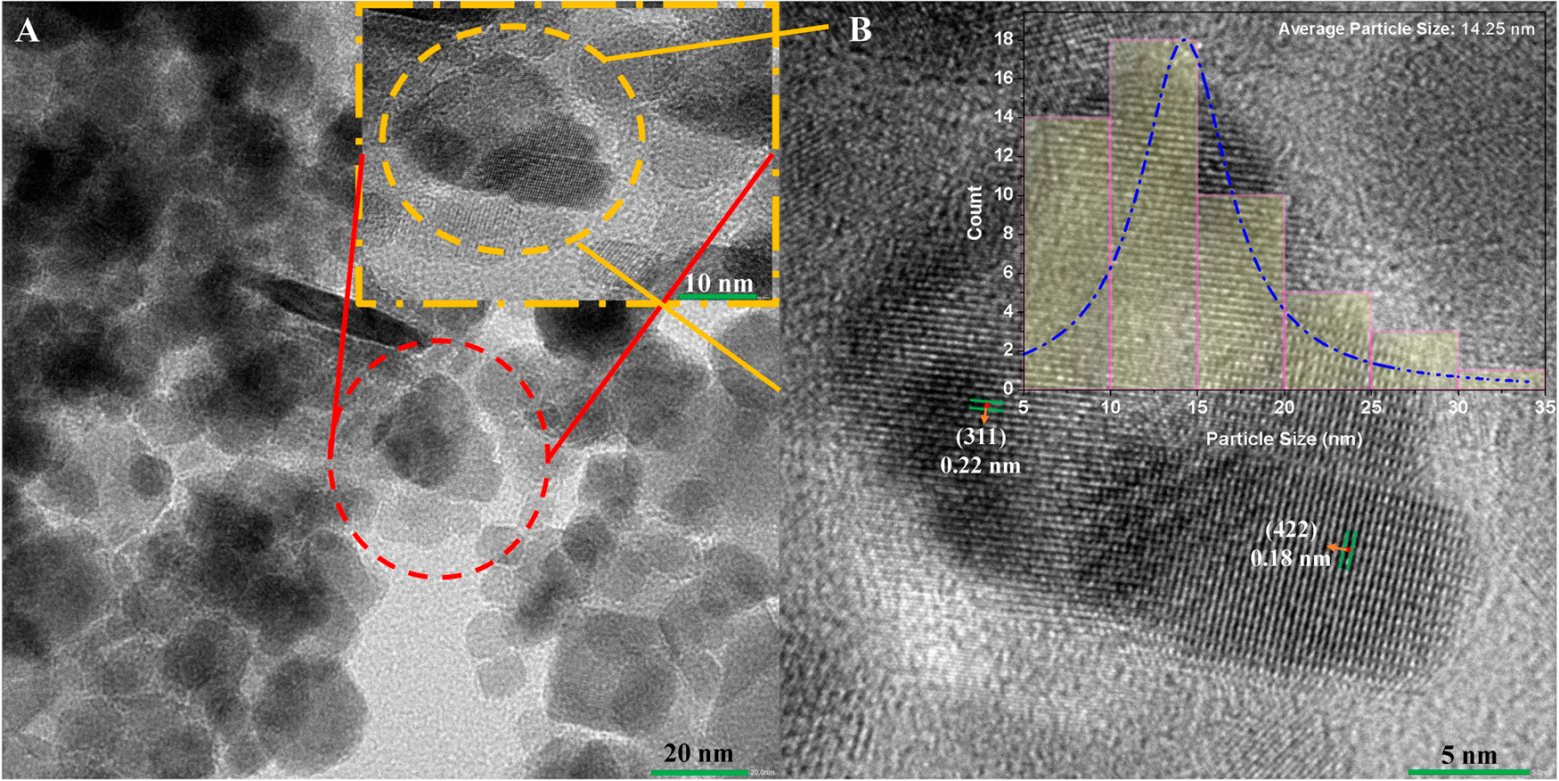
(A) TEM micrograph 20 nm (inset 10 nm) and (B) HR-TEM micrograph at 5 nm (inset average particle size distribution) of Fe_3_O_4_ NPs.

The X-ray photoelectron spectroscopy (XPS) analysis was conducted to confirm the elemental composition and oxidation states of Fe and O in the biogenically synthesized Fe_3_O_4_ NPs (Figure S2A–C). The survey spectrum revealed distinct peaks corresponding to Fe 2p and O 1s, verifying the presence of only Fe and O elements, consistent with the expected stoichiometry of Fe_3_O_4_. The high-resolution Fe 2p spectrum (Figure S2B) exhibits two characteristic peaks at binding energies of ∼711 eV (Fe 2p_3/2_) and ∼726 eV (Fe 2p_1/2_), accompanied by a satellite feature near ∼719.0 eV. These peaks confirm the coexistence of Fe^2+^ and Fe^3+^ oxidation states typical of magnetite (Fe_3_O_4_), distinguishing it from γ-Fe_3_O_4_, which lacks a prominent satellite peak around 719 eV. The Fe^2+^/Fe^3+^ coexistence arises from electron hopping between octahedral Fe sites, validating the inverse spinel structure of Fe_3_O_4_^25^. The deconvoluted O 1s spectrum (Figure S2C) further supports the formation of Fe_3_O_4_ by displaying four well-resolved peaks centered at 530.4, 527.7, 526.0, and 524.3 eV. The dominant feature at 530.4 eV corresponds to lattice oxygen (O^2–^) bonded to Fe in the spinel Fe–O–Fe framework. The second component at 527.7 eV is attributed to oxygen associated with Fe^2+^ sites or oxygen in slightly defective environments, reflecting the mixed-valence Fe^2+^/Fe^3+^ nature typical of magnetite. The peak at 526.0 eV is assigned to oxygen from Fe–O–H or substoichiometric Fe–O species arising from partial surface reduction or vacancy-induced defects, which enhance surface reactivity and catalytic potential. The smallest component at 524.3 eV represents chemisorbed or adsorbed oxygen species, possibly originating from residual phytochemicals or surface hydroxyl groups formed during the biogenic synthesis using the AN extract. This multicomponent deconvolution confirms the coexistence of both lattice and defect-related oxygen environments, consistent with mixed-valence Fe_3_O_4_ rather than Fe_2_O_3_ or FeO. Such surface-defect oxygen states are known to improve electron transfer and facilitate enzyme anchoring in biosensing applications.^25^ The quantitative XPS analysis (Table S2) revealed Fe and O atomic concentrations of 32.94 and 67.06%, giving an Fe:O ratio of 1:2.03, which closely matches theoretical Fe_3_O_4_ stoichiometry. These results corroborate the Fe 2p analysis and confirm the successful synthesis of phase-pure magnetite, consistent with prior reports.^26^

### Interfacial Studies

To characterize the fabricated nanoengineered electrodes (Fe_3_O_4_ NPs/SPE) and nanobioengineered electrode (ChO-Fe_3_O_4_ NPs/SPE), differential pulse voltammetry (DPV) was conducted using a three-electrode SPE-based setup. Initially, the pH optimization of the nanobioengineered electrode was performed by adjusting the pH from 5.7 to 8.0 in a 50 mM phosphate-buffered saline (PBS) solution containing 0.9% sodium chloride (NaCl) and 5 mM ferriferrocyanide (Fe­(CN)_6_)^3‑/4‑^. DPV analysis at a scan rate of 50 mV/s was employed to monitor changes in current across varying pH conditions. The results, illustrated in Figure 5A,B, demonstrated a notable increase in peak currents (*I*_p_) from pH 5.7 to 6.0, reaching a maximum at pH 6.0. Beyond this point, a decline in *I*_p_ was observed at pH 7.0, attributed to the reduced enzymatic efficiency of ChO under suboptimal pH conditions. However, a slight increase in *I*_p_ was recorded between pH 7.5 and 8.0, likely due to enhanced ionic conductivity of the PBS, which facilitates electron transfer despite the diminished catalytic activity of the enzyme.^27^ The drastic increase in *I*_p_ at pH 6.0 can be attributed to the optimal enzymatic activity of ChO, which exhibits a peak catalytic performance near its isoelectric point. At this pH, the enzyme maintains its native conformation. Deviations from this pH result in either partial denaturation or protonation/deprotonation of active site residues, reducing catalytic efficiency. Thus, pH 6.0 was found to be the optimal condition, which was used for a comparative performance analysis of the fabricated nanobioengineered electrode and the bare SPE. The DPV measurements were performed at pH 6.0 in a PBS solution at a scan rate of 50 mV/s to evaluate the electrodes’ efficiency for the electrochemical sensing of malathion pesticide. Initial DPV results taken for bare SPE, Fe_3_O_4_ NPs/SPE, and ChO-Fe_3_O_4_ NPs/SPE electrodes show an *I*_p_ of 0.31, 0.40, and 0.35 mA, respectively (Figure 5C,D). The observed increase in *I*_p_ from bare SPE to Fe_3_O_4_ NPs/SPE and ChO-Fe_3_O_4_ NPs/SPE highlights the enhanced electrochemical properties resulting from surface modifications. Among the modified electrodes, the Fe_3_O_4_ NP/SPE electrode exhibits the highest *I*_p_ compared to bare SPE and the ChO-Fe_3_O_4_ NP/SPE nanobioengineered electrode. This is attributed to the enhanced conductivity of Fe_3_O_4_ NPs, which enhance electron transfer and provide improved surface functionalization, promoting strong interactions between the electrode and mediator.^28^ However, immobilizing the ChO enzyme on the Fe_3_O_4_ NP/SPE electrode slightly decreases conductivity as the enzyme layer acts as an insulating barrier. Despite this, the synergistic effects of the enzyme’s physisorption onto the Fe_3_O_4_ NPs maintain better electron mobility than bare SPE, though the electron flow is somewhat reduced, leading to a lower *I*_p_ for the ChO-Fe_3_O_4_ NP/SPE electrode compared to the Fe_3_O_4_ NP/SPE electrode. The Fe_3_O_4_ NPs significantly enhance the electrode’s surface area, providing more active sites for electron transfer, thereby improving conductivity and increasing the current. However, in the ChO-Fe_3_O_4_ NP/SPE electrode, while the immobilization of ChO maintains a functional electrochemical interface, it slightly reduces conductivity due to the enzyme layer acting as an insulating barrier. Further, the utilization of Fe_3_O_4_ NPs extends beyond serving as a passive matrix, their mixed-valence states (Fe^2+^/Fe^3+^) enable fast redox mediation and enhanced charge transfer. The magnetically responsive nanofilm generated via electrophoretic deposition enhances enzyme loading and structural stability.^29^

**Figure 5:**
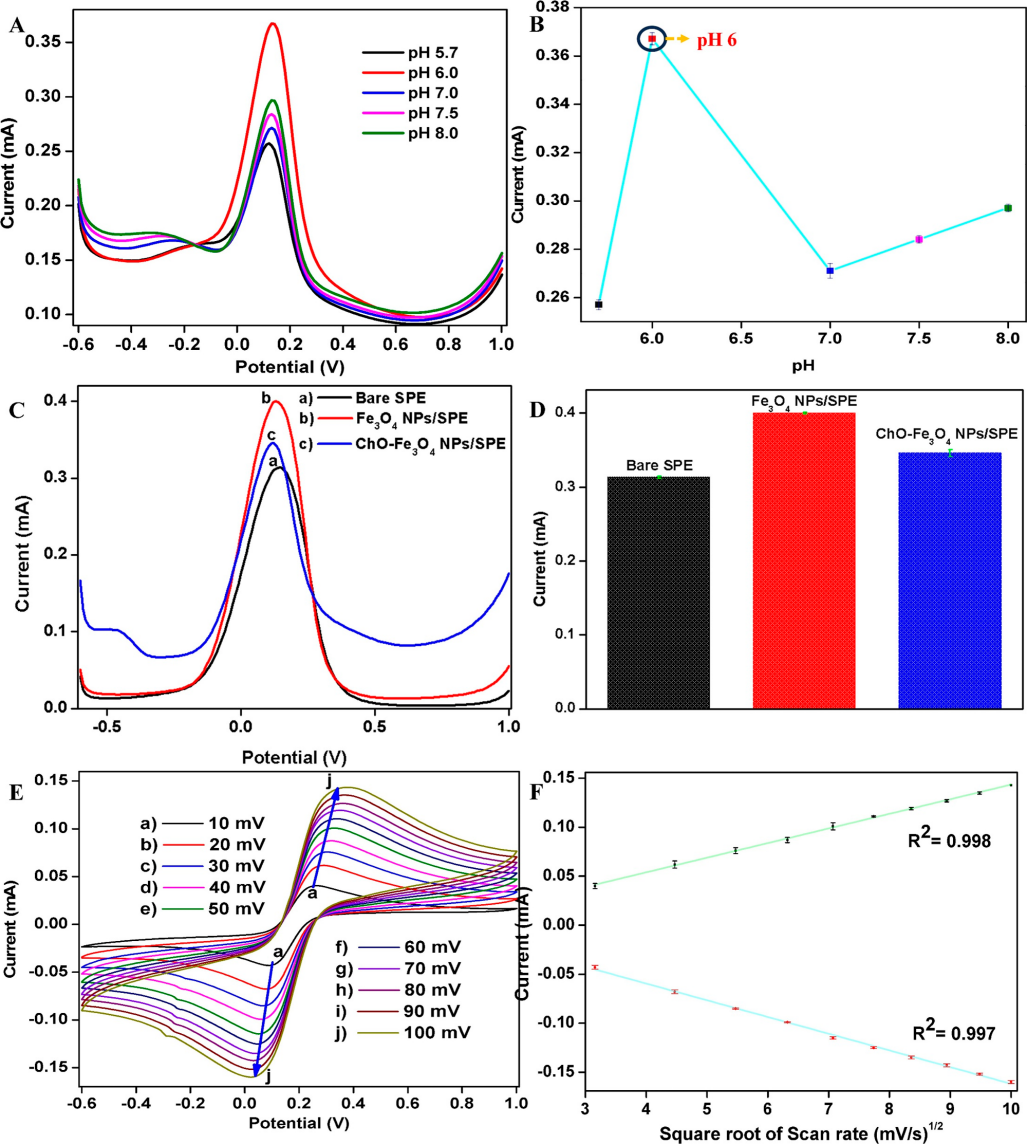
(A) ChO-Fe_3_O_4_ NP/SPE electrodes pH optimization using DPV, (B) pH vs *I*_p_ plot, (C) DPV responses of fabricated electrodes: (a) bare SPE, (b) Fe_3_O_4_ NPs/SPE, and (c) ChO-Fe_3_ *O*_4_ NPs/SPE, (D) *I*_p_ response of fabricated electrodes from DPV analysis in (C), (E) CV analysis of the ChO-Fe_3_ *O*_4_ NPs/SPE at different scan rates, and (F) plot of *I*_pa_ and *I*_pc_ vs square root of the scan rate showing a linear relationship.

### Interfacial Kinetics Studies

To investigate the interfacial kinetics of the fabricated ChO-Fe_3_O_4_ NP/SPE nanobioengineered electrodes prior to their use in the electrochemical biosensing of malathion, a scan-rate-dependent analysis was performed by varying the scan rate between 10 and 100 mV/s. This analysis aimed to explore the influence of scan rate on the current response, providing valuable insights into the reaction kinetics to understand the reversibility of the system. It was found that with an increase in the scan rate, the anodic peak current (*I*_pa_) increased, while the cathodic peak current (*I*_pc_) showed a decreasing trend (Figure 5E). Moreover, a positive shift in the anodic peak potential (*E*_pa_) and a negatively shifted cathodic peak potential (*E*_pc_) indicated the dominance of different reaction mechanisms at varying scan rates and potentials.^30^ To further evaluate the reaction mechanism, the *I*_pa_ and *I*_pc_ at each scan rate were plotted against the square root of the scan rate (*v*), as shown in Figure 5F. This approach assessed whether the reaction adhered to the linear relationship characteristic of diffusion-controlled processes, as described by eq 4. The observed linear relationship between the peak currents (*I*_pa_/*I*_pc_) and the square root of the scan rate confirmed that the reaction occurring at the nanobioengineered electrode interface followed a quasi-reversible mechanism. Minor deviations from perfect linearity were consistent with the quasi-reversible nature of the process, further validating the obtained results.^30^

4 $$I_{p}\propto \surd \nu$$

The interfacial kinetic analysis of the ChO-Fe_3_O_4_ NP/SPE nanobioengineered electrode revealed critical electrochemical parameters, including the charge-transfer rate constant (*K*_s_; eq 5), diffusion coefficient (D; eq 6), and surface concentration (*ϒ*; eq 7). The calculated values for *K*_s_, D, and ϒ are 0.54 s^–1^, 2.80 × 10^–2^ cm^2^ s^–1^, and 2.218 × 10^–7^ mol cm^–2^, respectively. Although slightly below 1 s^–1^, it still reflects rapid kinetics, in contrast to much slower rates characterized by values around 10^–4^ s^–1^.^31^ The value of *D*, determined using a modified Randles–Sevcik equation, was significantly higher than typical values (10^–5^ to 10^–6^ cm^2^ s^–1^) reported in the literature.^32^ This suggests highly efficient diffusion at the electrode surface, which supports rapid reaction kinetics and contributes to enhanced sensitivity. Additionally, the ϒ was calculated using the Brown–Anson model.^33^ The relatively low ϒ value reflects a limited number of electroactive sites, likely influenced by the immobilization of the ChO enzyme on the Fe_3_O_4_ NP/SPE interface. Despite this limitation, the electrode exhibits efficient interfacial behavior overall. The high *K*_*s*_ value highlights its capability for effective electron transfer, which is critical for malathion detection. Similarly, the substantial *D* emphasizes rapid kinetics, enabling diffusion-controlled processes at the interface to occur efficiently. While the low ϒ indicates a restriction in the availability of electroactive sites, it does not hinder the electrode’s overall electron transfer efficiency. These results collectively indicate that the reaction mechanism at the electrode interface is predominantly diffusion-controlled, making this electrode design highly suitable for the sensitive and specific detection of malathion. Further details on the variables and corresponding values used to solve eqs 5–7 can be found in the Supporting Information (Section S6).

5 $$K_{s}=\frac{mnF\nu }{RT}$$

6 $$I_{p}=\left(2.69\times 10^{5}\right)\surd \alpha AD^{1/2}C\nu^{1/2}$$

7 $$I_{p}=\frac{n^{2}F^{2}ϒA\nu }{4RT}$$

### Electrochemical Biosensing of Malathion

The integration of ChO with the Fe_3_O_4_ nanointerface enables a detection mechanism fundamentally different from that of conventional AChE-based inhibition assays. Here, we propose that the biosensing process is driven by oxidation events involving ChO, the Fe_3_O_4_ nanofilm, and the Fe­(CN)_6_^3–/4‑^ mediator rather than by inhibition of an enzyme. The biogenic Fe_3_O_4_ nanofilm, synthesized using AN extract, not only provides a biocompatible microenvironment for enzyme immobilization but also introduces mixed-valence (Fe^2+^/Fe^3+^) surface redox centers that accelerate electron transfer between the enzyme’s flavin cofactor and the electrode. The electrophoretic deposition method yields a compact and uniform Fe_3_O_4_ layer with strong adhesion, improving charge transport and long-term structural stability. Together, this synergistic enzyme–NP interface forms the basis of a noninhibitory, oxidation-driven biosensing concept, representing an alternative approach to traditional organophosphate sensors. The following section presents electrochemical evidence supporting this mechanism through chronoamperometry and differential pulse voltammetry measurements. Before initiating the electrochemical biosensing of malathion using the ChO-Fe_3_O_4_ NP/SPE nanobioengineered electrode, the response time of the system was evaluated through chronoamperometry after introducing 10 μL of a 60 μM malathion solution over 120 s at a constant potential (E) of 0.121 V, with current measurements recorded at 0.1 s intervals (Figure 6A). The current response reached 90% of its maximum value within 28.7 s, after which it stabilized and remained nearly constant for up to 120 s.

**Figure 6:**
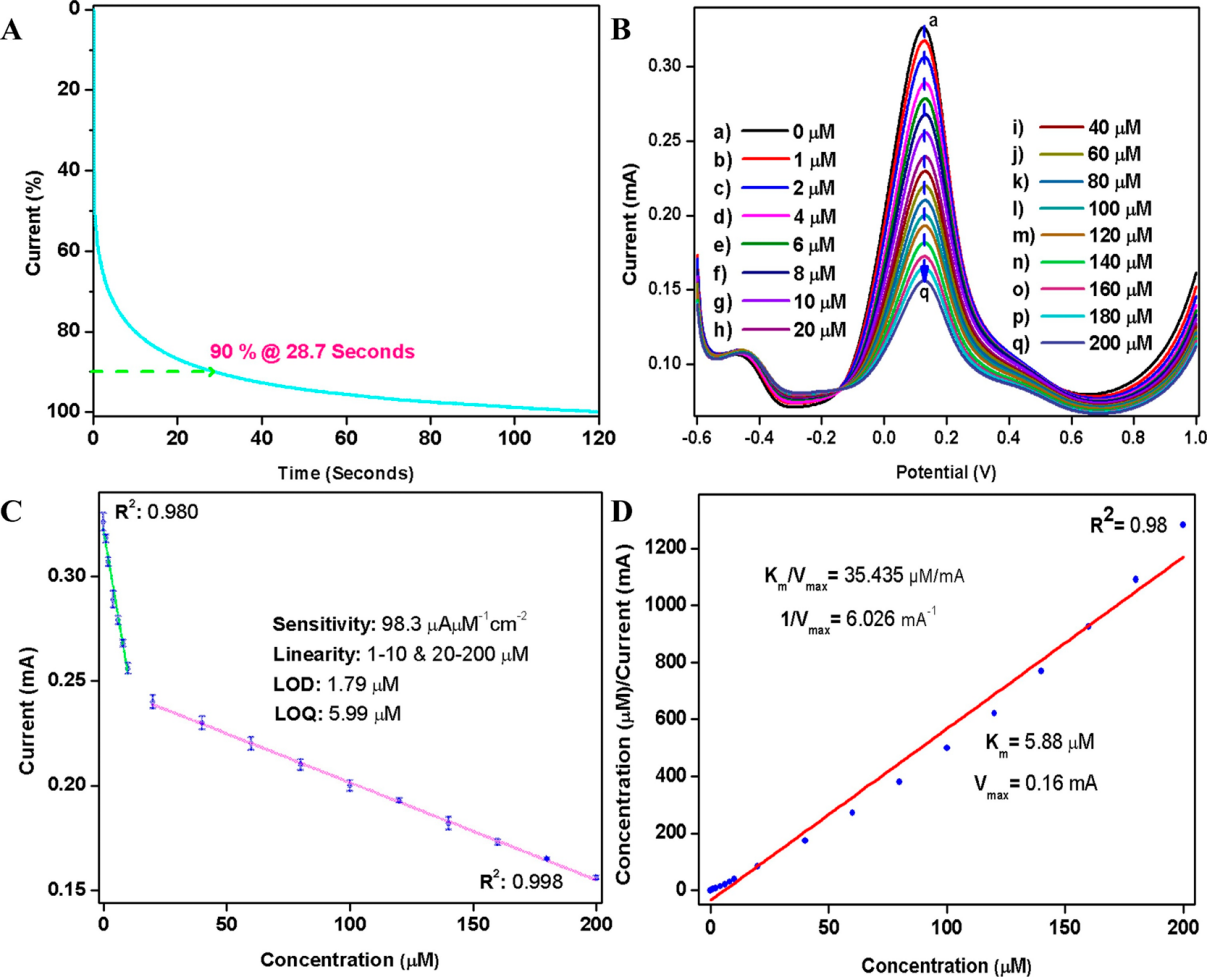
Nanobioengineered ChO-Fe_3_ *O*_4_ NP/SPE electrodes: (A) Response time analysis, (B) detection at different malathion levels, (C) calibration curve of I_p_ vs malathion concentration, and (D) Hanes–Woolf plot: determining *K*_m_ and *V*_max_.

The normal malathion concentration ranges in water, soil, and agricultural products are reported to be between 0.01 and 0.1 μM, 1 and 50 μM, and 0.01 and 1 μM, respectively, and concentrations beyond this are considered abnormal or potentially harmful.^34–36^ This provided a crucial benchmark for calibrating the biosensor and assessing its sensitivity and accuracy in detecting malathion. Based on the identified benchmarks, malathion concentration ranges of 1–200 μM were selected to calibrate and evaluate the performance of the fabricated ChO-Fe_3_O_4_ NP/SPE electrode. The analysis was conducted under optimized conditions using PBS solution at pH 6.0 containing 0.9% NaCl and 5 mM (Fe­(CN)_6_)^3–/4–^. A delay of 28.7 s was allowed after each addition of malathion to ensure the electrode had sufficient time to respond effectively to the analyte.

The analysis results (Figure 6B) reveal that the *I*_p_ decreases linearly with increasing malathion concentration, specifically within the 1–10 and 20–200 μM ranges (Figure 6C). This linear relationship underscores the high reliability and accuracy of the fabricated biosensors for malathion detection. Further analysis of the data presented in Figure 6B,C facilitated the calculation of the electrode’s limit of detection (LOD), limit of quantification (LOQ), and sensitivity using eqs 8, 9, and 10, respectively. The standard deviation (SD) of the blank sample was determined to be 6.95 × 10^–3^, the slope (*S*) of the calibration curve was 4.16 × 10^–3^, and the active area (*A*) of the electrode was taken as 7.07 × 10^–2^ cm^2^. By substitution of these values into the respective equations, the LOD, LOQ, and sensitivity were calculated to be 1.79 μM, 5.99 μM, and 98.3 μA μM^–1^cm^–2^, respectively. These results demonstrate the electrode’s high sensitivity and suitability for accurate and quantitative detection of malathion over the studied concentration ranges.

8 $$LOD=\frac{3\times SD}{S}$$

9 $$LOQ=\frac{10\times SD}{S}$$

10 $$Sensitivity=\frac{S}{A}$$

The obtained results demonstrate that the fabricated biosensor is highly effective for detecting environmental contamination, as it can accurately detect and quantify malathion concentrations within and beyond environmentally safe ranges in soil and food. The low LOD and LOQ values highlight their ability to detect malathion at trace levels, ensuring the reliable monitoring of contamination. Furthermore, the significant change in signal per unit concentration of malathion, reflected by its high sensitivity, indicates an optimal balance between sensitivity and detection limits, enhancing its applicability for precise environmental monitoring, as summarized in Table 1. The biosensor achieves high sensitivity and performs competitively with the sensors summarized in Table 1. Its wide linear detection range (1–200 μM) further adds to its versatility, making it suitable for a variety of practical applications. While the LOD of this biosensor is slightly higher than that of some previously reported sensors, it remains competitive and sufficient for real-world malathion monitoring. This trade-off is offset by its favorable combination of sensitivity, broad linear range, and ease of fabrication using eco-friendly Fe_3_O_4_ NPs. The ChO-Fe_3_O_4_ NP/SPE biosensor combines a high detection accuracy with practical usability, making it a dependable option for environmental monitoring. Unlike conventional chemically synthesized Fe_3_O_4_, which often requires surfactants or high-temperature stabilization, the green-synthesized Fe_3_O_4_ NPs here exhibited long-term structural and electrochemical stability, as evidenced by minimal degradation of DPV current (<2% after 2 months). This performance is comparable to that of the chemically synthesized systems reported in Table 1.

**Table 1:** Comparison of Previous Work with the Present Investigation^a^

s.no	material	linear range (μM)	sensitivity (μAμM^–1^cm^–2^)	LOD (μM)	ref
1	ChO-Fe_3_O_4_ NPs/SPE	1–10	98.3	1.79	**present work**
		20–200			
2	Nafion/AChE/CS-PB-MWCNTs-HGNs/Au	0.00005–0.07	0.0051	0.00005	^37^
3	poly(TTP)/AChE/GCE	15–1644	0.18	0.004	^38^
4	AChE/Pan/PPy/MWCNTs/GCE	0.03–1.51		3.03	^39^
		3.03–75.8			
5	Eu^3+^–(Batho)_2_ probe	0.5–7		0.92	^40^
6	AChE/Pan/PPy/MWCNTs/GCE	0.03–1.51		3.03	^39^
		3.03–75.8			
7	AChE/CPBA/AuNPs/RGO/GCE	1.51–30.3	0.016	1.51	^41^
		60.6–303.0			

a Abbreviations: bathophenanthroline (Batho); europium (Eu^3+^); triphenylphosphonium (TTP); acetylcholinesterase (AChE); carboxyphenylboronic (CPBA); reduced graphene oxide (RGO); gold nanoparticles (AuNPs); multiwalled carbon nanotubes (MWCNTs); chitosan (CS); Prussian blue (PB); hollow gold nanospheres (HGNs); gold (Au); glassy carbon electrode (GCE); polyaniline (Pan); polypyrrole (PPy).

To elucidate the mechanism of the ChO enzyme immobilized on the Fe_3_O_4_ NP/SPE substrate, enzyme kinetics was assessed by determining the Michaelis–Menten constant (*K*_m_) and maximum velocity (*V*_max_). These values, calculated from a Hanes–Woolf plot (Figure 6D) that plots the analyte concentration against the ratio of analyte concentration to current (concentration/*I*_p_), were found to be 5.88 μM and 0.16 mA, respectively. This linearization method determines *K*_m_ and *V*_max_ by fitting a straight line, where the slope corresponds to 1/*V*_max_ and the y-intercept provides *K*_m_/*V*_max_. The obtained *K*_m_ value suggests a moderate binding affinity of the enzyme for malathion, requiring relatively higher concentrations of malathion to achieve half of the maximum reaction velocity. This indicates effective malathion binding and conversion, although at higher analyte concentrations. Furthermore, the reduced *V*_max_ reflects the limited catalytic efficiency of the immobilized enzyme, likely due to constraints introduced by the immobilization process. These limitations slightly restrict enzyme activity but do not impede its functionality for malathion detection. It is important to note that the kinetic parameters derived here represent apparent solid-state values governed by interfacial diffusion rather than bulk enzymatic turnover; therefore, direct comparison with free-solution ChO kinetics is not meaningful. As illustrated in Figure 7A, a plausible oxidation-based mechanism is proposed for malathion sensing using the ChO-Fe_3_O_4_ NP/SPE nanobioengineered platform. Although ChO is classically specific for the oxidation of choline to betaine aldehyde with concomitant hydrogen peroxide (H_2_O_2_) generation,^42,43^ it has been reported that flavin-containing oxidases, including ChO, can exhibit weak promiscuous activity toward structurally analogous ester and phosphoester substrates.^44,45^ Owing to the presence of a choline-like ester functionality within the malathion molecule, ChO immobilized on Fe_3_O_4_ may contribute to partial oxidative transformation of malathion or its hydrolysis intermediates and to the generation of H_2_O_2_ as an electroactive byproduct, although this requires future biochemical validation.^46^ In addition, surface-active Fe_3_O_4_ nanostructures, possessing mixed Fe^2+^/Fe^3+^ redox centers, can participate in redox mediation and reactive oxygen species generation, further amplifying the oxidation current. The resulting H_2_O_2_ molecules undergo electrochemical oxidation at the Fe_3_O_4_-modified electrode, yielding measurable anodic signals, even at trace analyte concentrations. The synergistic interaction between the enzyme’s FAD cofactor and the Fe_3_O_4_ redox interface facilitates rapid electron transfer, accounting for the short response time and high sensitivity observed experimentally.

**Figure 7:**
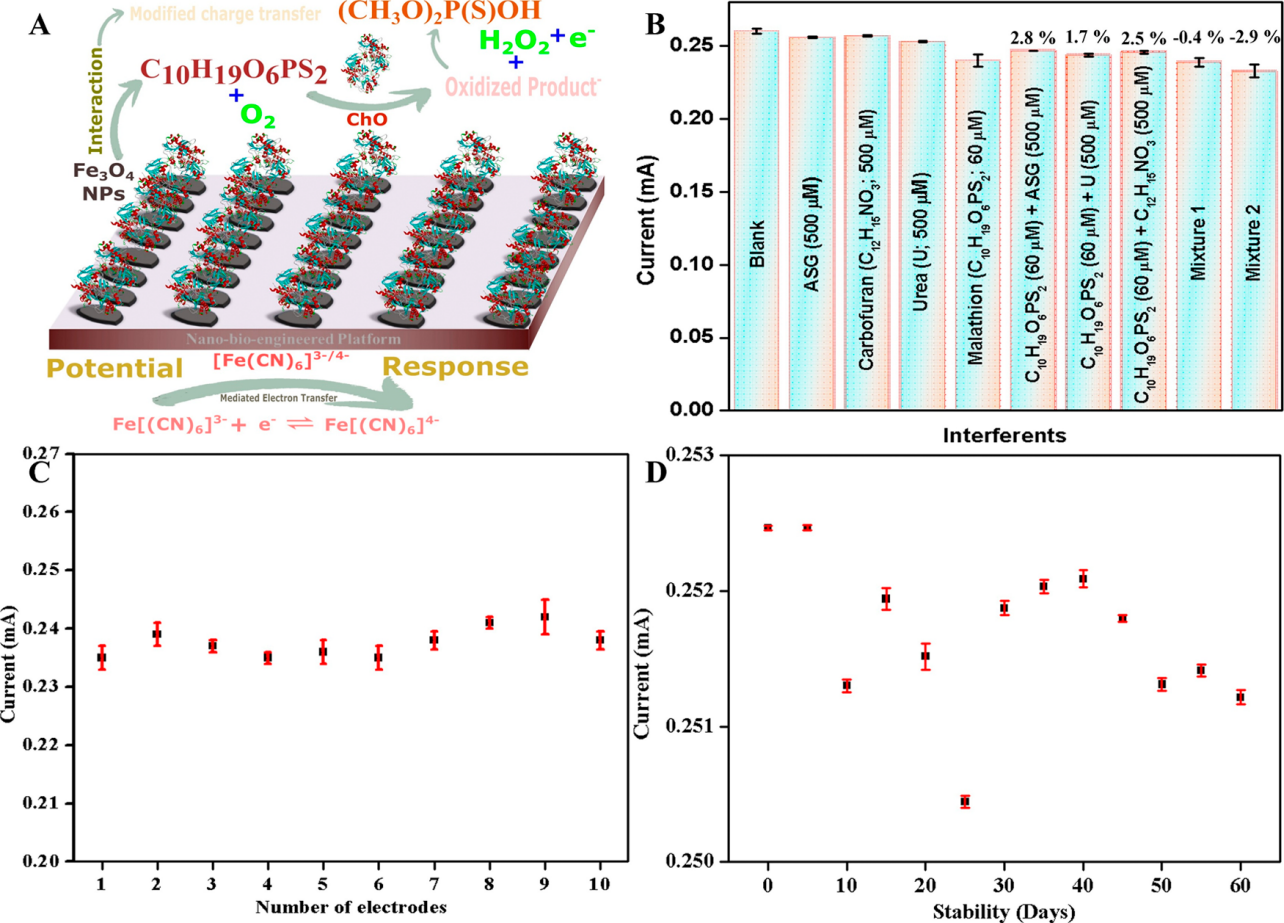
ChO-Fe_3_ *O*_4_ NP/SPE nanobioengineered electrode: (A) Mechanism of malathion biosensing, (B) interference studies, (C) reproducibility test, and (D) stability test.

At higher concentrations of malathion, the system exhibits a gradual decrease in the current response. This behavior can be explained by the electrode entering a mass-transport-limited regime. As malathion and its reaction products accumulate near the ChO–Fe_3_O_4_ layer, they form a concentration–polarization region that restricts the diffusion of the Fe­(CN)_6_^3–/4‑^ redox mediator to and from the electrode surface. This diffusion barrier reduces the peak current, even as the bulk analyte concentration increases. Similar decreasing-current responses have been reported in quasi-reversible and diffusion-limited electrochemical systems.^46,47^ Thus, the linear decrease in current with increasing malathion concentration (Figure 6C) is attributed to mass-transport limitations at the modified electrode rather than to a loss of catalytic activity.

The fabricated ChO–Fe_3_O_4_ NP/SPE electrode was evaluated for its selectivity through interference studies (Figure 7B). Initially, a blank DPV response was recorded, followed by individual DPV readings for 500 μM concentrations of common interferents, including carbofuran, urea, and ammonium salt of glyphosate (ASG). Additional DPV measurements were taken for 60 μM malathion separately and then in combination with each interferent (500 μM). Finally, a DPV response was recorded for a mixture containing all of the interferents (500 μM each) along with malathion (60 μM). Subsequently, to further validate the electrode’s selectivity under more complex and realistic environmental conditions, two mixtures were prepared. The first, termed mixture 1, contained carbofuran, urea, and ASG at 500 μM each, along with 60 μM malathion. The second, referred to as mixture 2, incorporated a broader range of potential interferents to simulate agricultural and environmental matrices, including carbofuran, urea, ASG, Li^+^, Na^+^, K^+^, Cs^+^, Mg^2+^, Ca^2+^, Zn^2+^, Pb^2+^, Ni^2+^, Fe^2+^, Cr^3+^, Co^2+^, Cd^2+^, Al^3+^, Ag^+^, humic acids, Tween-80 (surfactant), clothianidin, and carbendazim, each at 1000 μM concentration, combined with 60 μM malathion. The results confirmed that the electrode exhibits an excellent selectivity for malathion detection. The current response showed minimal variation for blank and individual interferent samples, while the presence of malathion caused a significant and distinct response. Even in the presence of mixtures 1 and 2, the electrode retained a clear and well-defined malathion signal, indicating its capability to discriminate against structurally and chemically diverse interferents present at concentrations more than an order of magnitude higher than the target analyte. This selectivity was maintained even in the presence of all the interferents, with the percentage change in current due to interferents ranging from −2.9% to 2.8%, ensuring minimal interference for malathion detection. The average relative standard deviation (RSD) for the interference studies was calculated to be 0.7%, further demonstrating the sensor’s reliability and resistance to interference. Thus, the ChO–Fe_3_O_4_ NP/SPE electrode demonstrates robust selectivity and stability, comparable to high-performance malathion sensors reported in the literature, even under multicomponent interference conditions. Further, to ensure optimal electrochemical performance of the nanobioengineered electrode, the loading amount of ChO on the Fe_3_O_4_ NP/SPE substrate was optimized. Since enzyme concentration directly influences catalytic efficiency and current response, three different loadings of ChO solution (5 μL, 10 μL, and 20 μL) were tested using DPV (Figure S3). The ChO enzyme (from Alcaligenes sp., ≥ 10 units) contained approximately 5 units of activity in 20 μL, with lower volumes corresponding to proportionally reduced enzyme activities. Among these, the electrode modified with 10 μL of the enzyme solution exhibited the highest and most stable current response. Lower loading (5 μL) resulted in reduced catalytic activity due to insufficient enzyme coverage, whereas excessive loading (20 μL) led to diffusion hindrance and decreased electron transfer efficiency caused by surface crowding. Hence, 10 μL was selected as the optimized ChO loading for all subsequent electrochemical measurements, providing the best balance among catalytic activity, signal stability, and reproducibility.

Reproducibility tests (Figures 7C and S4A) were conducted by fabricating 10 identical ChO–Fe_3_O_4_ NP/SPE electrodes and recording DPV responses in the presence of 60 μM malathion. The average RSD for reproducibility was determined to be 0.24%, highlighting the sensor’s strong reproducibility and consistent performance across multiple electrodes. Additionally, stability tests (Figures 7D and S4B) were performed over 2 months with DPV measurements taken at 5-day intervals using 60 μM malathion. The results showed an average RSD of 0.02%, indicating that the electrode maintained high stability throughout the testing period. RSD values were calculated from *n = 3* independent measurements at each interval, reported as the mean ± SD. The low RSD values arise from the uniformity of the Fe_3_O_4_ EPD layer and stable physisorption of ChO over 60 days. These findings demonstrate that the fabricated biosensor offers outstanding electro-analytical performance for malathion detection. This confirms that even without covalent cross-linking, the physically adsorbed enzyme remains stable owing to electrostatic and hydrogen-bonding interactions with the hydroxylated Fe_3_O_4_ surface, ensuring a negligible loss in catalytic performance over prolonged storage. The combination of high sensitivity, broad detection range, excellent stability, and reproducibility establishes the ChO–Fe_3_O_4_ NP/SPE electrode as a highly effective tool for environmental monitoring, providing significant advantages over existing malathion sensors.

Molecular docking analysis (Figure 8A) was carried out to further substantiate the experimental findings and elucidate the binding orientation of malathion within the ChO-active pocket. The best docking conformation exhibited a binding affinity of −4.9 kcal/mol, suggesting spontaneous complex formation. The ligand formed multiple stabilizing interactions with catalytic residues, notably Glu114, Tyr119, Lys118, Arg345, and Arg383 (Figure 8B), which are known to play essential roles in substrate oxidation and proton transfer in ChO catalysis.^42^ The presence of hydrogen bonding with Arg383 and Arg345 enhances substrate stabilization, while hydrophobic contacts with Leu343 and His412 maintain structural alignment of the malathion molecule within the FAD-binding domain (Figure 8C).^48^ Furthermore, electrostatic interactions with Glu505 likely contribute to the charge complementarity and optimal orientation of the phosphoryl oxygens of malathion during binding. The combination of polar and hydrophobic interactions indicates that malathion adopts a productive pose in the active cleft, possibly competing with the natural substrate (choline). This supports the hypothesis that malathion binding perturbs the electron transfer pathway within ChO, as observed from the altered differential pulse voltammetry (DPV) profiles. Thus, the docking results provide molecular evidence of direct and specific binding, corroborating the enzymatic and electrochemical data. The moderate binding affinity (−4.9 kcal/mol) further aligns with our kinetic findings (*K*_m_ = 5.88 μM), reflecting noncovalent but stable substrate–enzyme interaction. This integrative approach, combining docking with electrochemical and kinetic analyses, establishes a coherent mechanistic model in which malathion interacts directly at the ChO catalytic site, modulating its oxidative activity.

**Figure 8:**
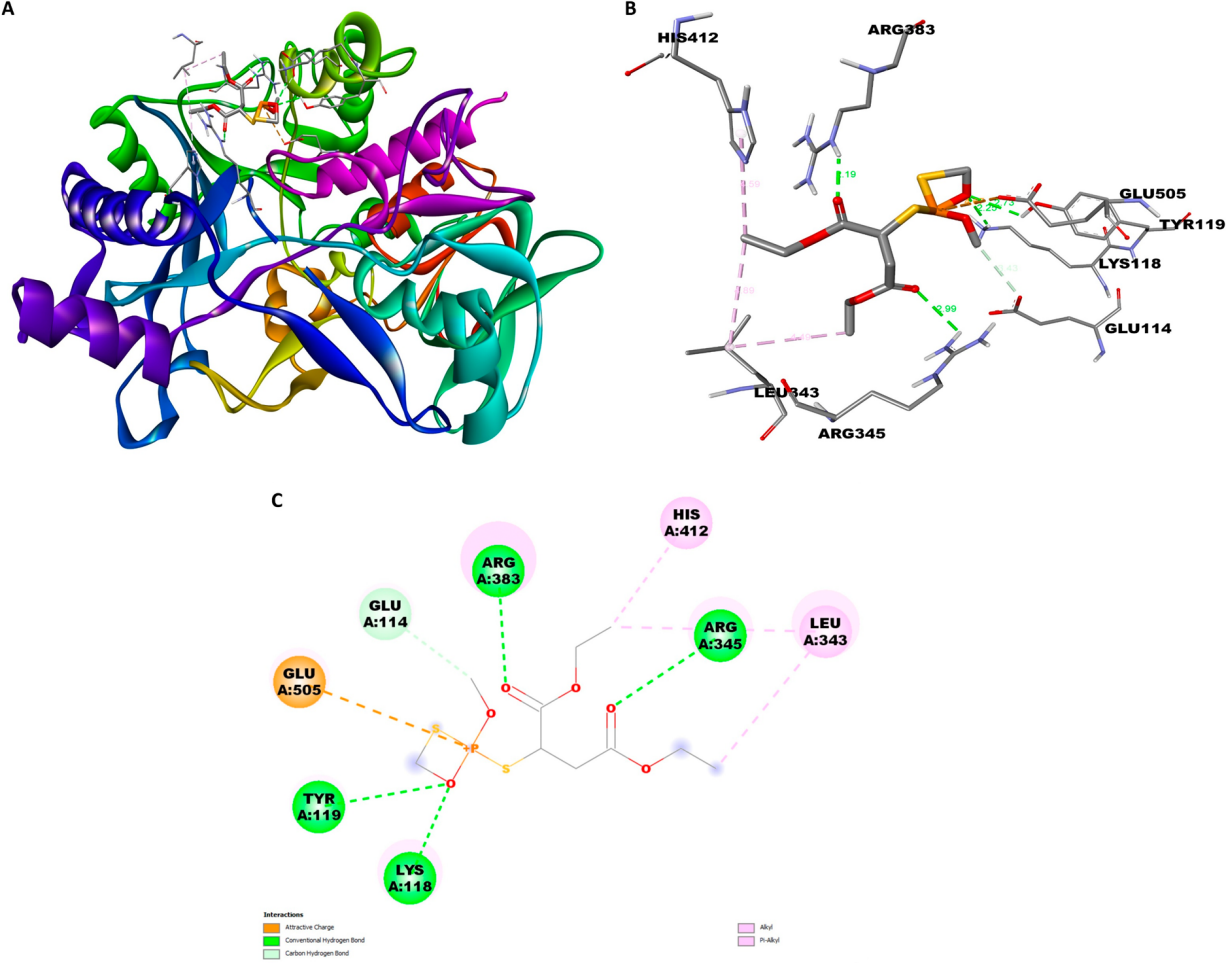
Molecular docking analysis of the malathion–ChO complex: (A) Overall 3D conformation of ChO showing malathion (stick representation) docked in the active pocket, (B) close-up view of the binding site highlighting hydrogen bonds and hydrophobic interactions with active site residues, and (C) 2D interaction map illustrating key residues involved in binding: Glu114, Tyr119, Lys118, Arg345, Arg383, His412, Leu343, and Glu505. The color-coded interactions represent hydrogen bonds (green), carbon–hydrogen interactions (light-green), and electrostatic contacts (orange). Docking scores (−4.9 kcal/mol) indicate a stable complex, corroborating direct molecular recognition between malathion and ChO-active site residues.

For comparison with conventional methods, HPLC analysis of malathion at 200 μM was attempted; however, the signal fell below the instrumental detection limit, as shown in Figure S5. This highlights one of the advantages of electrochemical biosensing, which enables rapid detection at submicromolar levels without time-consuming separation or derivatization steps. To assess the practical applicability of the ChO–Fe_3_O_4_ NP/SPE electrode for detecting malathion in real soil samples, a series of experiments involving spiked soil sample analysis was conducted. In these experiments, known concentrations of malathion were added to soil to evaluate the electrode’s performance.^49^ Initially, 2 g of soil was weighed and mixed with 50 mL of Milli-Q water. The mixture was then continuously stirred and heated to 120 °C for 10 min. After cooling, the solution was subjected to probe sonication (10 min at 50% duty cycle) to enhance the release of adsorbed analytes from the soil matrix. Subsequently, five sets of soil extracts were prepared: one containing only soil and four others spiked with malathion at concentrations of 10, 60, 100, and 140 μM. Each extract was diluted 5-fold with Milli-Q water before analysis. DPV was employed to record the electrochemical response currents for both the standard malathion solutions and spiked soil extracts. As shown in Figure 9, the *I*_p_ values recorded for the spiked soil samples closely matched those of the corresponding standard solutions, demonstrating the reliability of the electrode. Recovery analysis, detailed in Table S3 of Supporting Information S7, further validated the electrode’s accuracy. The recovery percentages for malathion concentrations of 10, 60, 100, and 140 μM were 96.1%, 93.9%, 98.4%, and 97.2%, respectively, highlighting the sensor’s high accuracy. Moreover, the RSD values, ranging from 0.009% to 0.87%, confirmed the precision and consistency of the detection method. These results demonstrate that the ChO–Fe_3_O_4_ NP/SPE electrode is highly effective and reliable for detecting malathion in real soil samples. The high recovery rates and low RSD values validate the electrode’s potential for practical applications in environmental monitoring and pesticide residue detection, establishing it as a valuable tool for field-level analysis. In addition, the negligible current variation observed for mixture 2 (containing humic acids and metal ions) supports the idea that common soil components do not significantly interfere with sensor performance.

**Figure 9:**
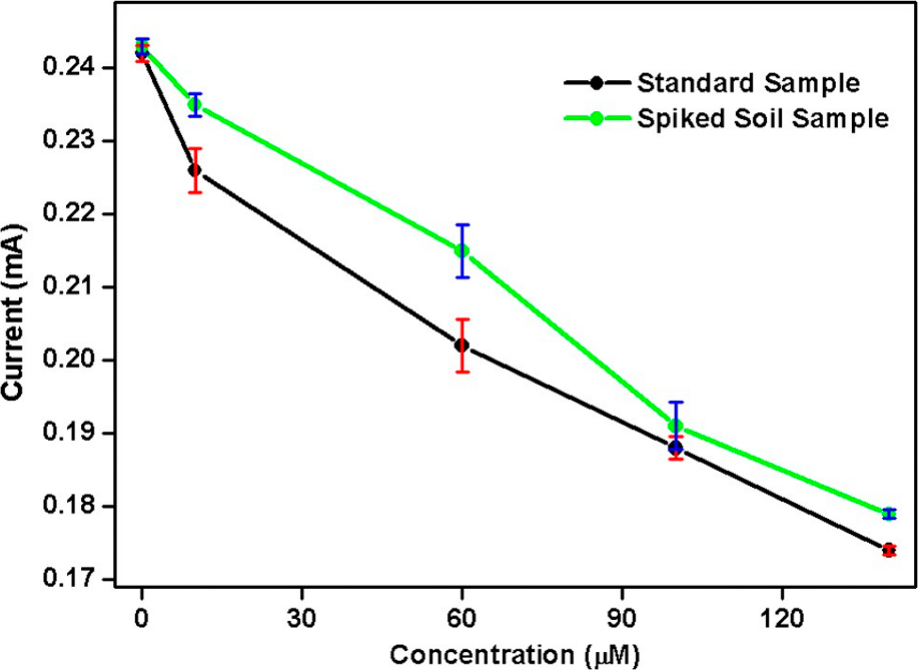
Real sample analysis in spiked soil samples for malathion detection.

## Conclusions

In this work, a nanobioengineered platform was developed utilizing Fe_3_O_4_ NPs synthesized via an eco-friendly, plant-mediated approach to fabricate the ChO–Fe_3_O_4_ NP/SPE biosensor for malathion detection. The synthesized Fe_3_O_4_ NPs exhibited exceptional structural and electrochemical properties, providing a stable and efficient matrix for enzyme immobilization. The resulting biosensor demonstrated high sensitivity, a low LOD of 1.79 μM, and a broad linear detection range (1–200 μM), making it highly competitive among existing pesticide sensors. The sensor’s excellent selectivity, stability, and reproducibility further enhance its reliability for real-world applications. To validate its practical utility, a real-sample analysis was conducted using spiked soil samples, achieving high recovery rates (93.9–98.4%), which confirmed the sensor’s effectiveness for real-world environmental monitoring. The unique combination of a green-synthesized Fe_3_O_4_ nanofilm assembled via electrophoretic deposition and the ChO-based oxidative detection principle distinguishes this study from prior inhibition-type biosensors, providing a sustainable and mechanistically innovative route for pesticide detection. These results underscore the potential of this eco-friendly biosensing approach for field-deployable applications in food safety and environmental surveillance. This study lays a foundation for scalable and sustainable biosensing solutions, contributing to safer agricultural practices and improved public health monitoring.

## Supplementary Material



## Notes

Materials and instrumentation used in the study, XRD crystallite size calculation, FTIR data of thin films on SPE, XPS analysis, details of kinetics studies variables, enzyme loading, reproducibility, stability, and real sample analysis (PDF)

## Notes

No ethical permission is required for this work.

## Notes

All the authors give their consent to participate in this work as authors.

## Notes

All the listed authors in this manuscript give their consent to publish the manuscript.

## Notes

The data will be made available upon request by the authors.
