# Supplementary material for: Highly Efficient and Selective Biosensing of Malathion Utilizing Bioengineered Nanoplatform Based on Fe3O4
Source: Langmuir. 2026 Jan 7;42(7):5533–46. doi: 10.1021/acs.langmuir.5c05560 (PMC12937094; doi:10.1021/acs.langmuir.5c05560)
Supplement: Supplementary file 1 [file la5c05560_si_001.pdf]

Supporting Information

Highly Efficient and Selective Biosensing of Malathion utilizing Bioengineered Nanoplatform based on Fe_3O_4

Kshitij RB Singh[1*], Pooja Singh[2], Shyam S. Pandey[1*]

[1]Graduate School of Life Science and Systems Engineering, Kyushu Institute of Technology, 2-4 Hibikino, Wakamatsu, Kitakyushu (808-0196), Japan.

[2]Department of Biotechnology, Indira Gandhi National Tribal University, Amarkantak, Madhya Pradesh (484886), India.

[*]Corresponding author (**S.S.P.**: shyam@life.kyutech.ac.jp; **K.R.B.S.**: krbs09@gmail.com)

Number of pages: 10

Number of figures: 05

Number of schemes: 0

Number of tables: 03

Table of Contents

S1. Materials....2
S2. Instrumentation 2
S3. XRD crystallite size calculation of Fe_3O_4 NPs....4
S4. FTIR Analysis 5
S5. XPS Analysis 6
S6. Details of Variables in Equations (Kinetics Studies)....7
S7. Enzyme Loading, Reproducibility, Stability, and Real Sample Analysis....8

S1. Materials

Iron(II) sulfate heptahydrate ($FeSO_4.7H_2O$; MW: 278.01 g mol^{-1}), Iron(III) chloride ($FeCl_3$; MW: 162.20 g mol^{-1}), potassium hexacyanoferrate (II) trihydrate ($C_6FeK_4N_6 \cdot 3H_2O$; MW: 422.39 g mol^{-1}; Product cat .no.: 1.93686.0521), potassium hexacyanoferrate (III) ($C_6FeK_3N_6$; MW: 329.25 g mol^{-1}; Product cat no.: 1.93667.0521), Malathion ($C_{10}H_{19}O_6PS_2$; MW: 330.36; Product cat no.: 36143-100MG), sodium chloride (NaCl; MW: 58.44 g mol^{-1}; Product cat no.: S9888), Choline oxidase from *Alcaligenes* sp. (≥10 units/mg solid; CAS No. : 9028-67-5; EC No.: 232-840-0; Product cat no.: C5896-50UN) and Whatman filter paper Grade-1, all were purchased from Sigma-Aldrich, USA. Sodium hydroxide (NaOH; MW: 40.00 g mol^{-1}; CAS no.: 1310-73-2) and ammonia liquor (NH_3; MW: 17.03 g mol^{-1}; CAS no.: Q16225) were obtained from Qualigens, Thermo Fisher Scientific, USA. Disodium phosphate ($Na_2HPO_4 \cdot H_2O$; MW: 177.99; CAS No.: 10028-24-7), and monosodium phosphate (NaH_2PO_4; MW: 119.98; CAS no.: 7558-80-7) were procured from Himedia, India. The ItalSens Carbon SPE (support material: polyester, WE: carbon, RE: silver; CE: carbon; WE geometric area: 0.0707 cm^2; Ø of the disc WE: 3 mm) was procured from PalmSens, Netherlands, for the electrophoretic deposition of prepared nanoparticles. Milli-Q water (18 Ω resistivity; Millipore, USA) was used in all the experimental works. In addition, the utilized phosphate buffer saline (PBS) in this work contains 0.9% saline NaCl and 5 mM of ferric ferrocyanide $(Fe(CN)_6)^{3-/4-}$. *Argyreia nervosa (AN)* leaves were collected from Indira Gandhi National Tribal University, Amarkantak, Madhya Pradesh, India, by Dr. Pooja Singh (one of the authors *of this work*).

S2. Instrumentation

Infrared spectra were recorded with a Nicolet iS5, Thermo Fisher Scientific, USA, in the spectral range 500-4000cm^{-1} of the synthesized nanoparticles. UV-visible spectroscopy was

performed by using UV-1800 Shimadzu, Japan, in the wavelength range of 200-600 nm to investigate NP's optical properties. XRD techniques examined the structure and crystalline size of Fe_3O_4 NPs by using an X-ray diffractometer (D8 Advance, Bruker, USA) with a Cu-Kα (λ = 1.5406 Å) in the 2θ angles ranging from 30 to 80°. Further, SEM and energy dispersive X-ray analysis (EDX) were performed to determine surface morphology and element confirmation of synthesized NPs by using Thermo Scientific, USA Quattro S., having an EDX attachment of EDS Ultradry. TEM analysis was performed for synthesized NPs by utilizing JEM-F200 (multi-purpose electron microscope, Acceleration voltage: 200kV), JEOL, Japan. For performing HPLC experiments, the following four instruments were used: Waters [600 Controller], GASTORR [AG-42], HITACHI [UV-Vis Detector L-7420], and HITACHI [D-2500 Chromato-Integrator]. Further, the electrochemical Malathion sensing studies of the fabricated ChO-Fe_3O_4 NPs/SPE nanobioengineered electrode were performed by EmStat4S electrochemical workstation of PalmSens, Netherlands, using three three-electrode system. This workstation is gifted by *Class One Systems S&T Pvt. Ltd.* for performing electrochemical studies in Pandey Laboratory at Kyushu Institute of Technology, Japan.

S3. XRD crystallite size calculation of Fe_3O_4 NPs

Table S1. *Crystallite size calculation.*

Parameters		Calculations		D (nm)	Average D (nm)
K	λ	Peak position 2θ (°)	FWHM β (°)		
0.94	1.5406	31.6	0.25	34.38	10.36
		35.7	1.26	6.88	
		45.6	1.58	5.67	
		55.2	5.46	1.71	
		57.3	1.35	6.99	
		63.1	1.48	6.53	

S4. FTIR Analysis

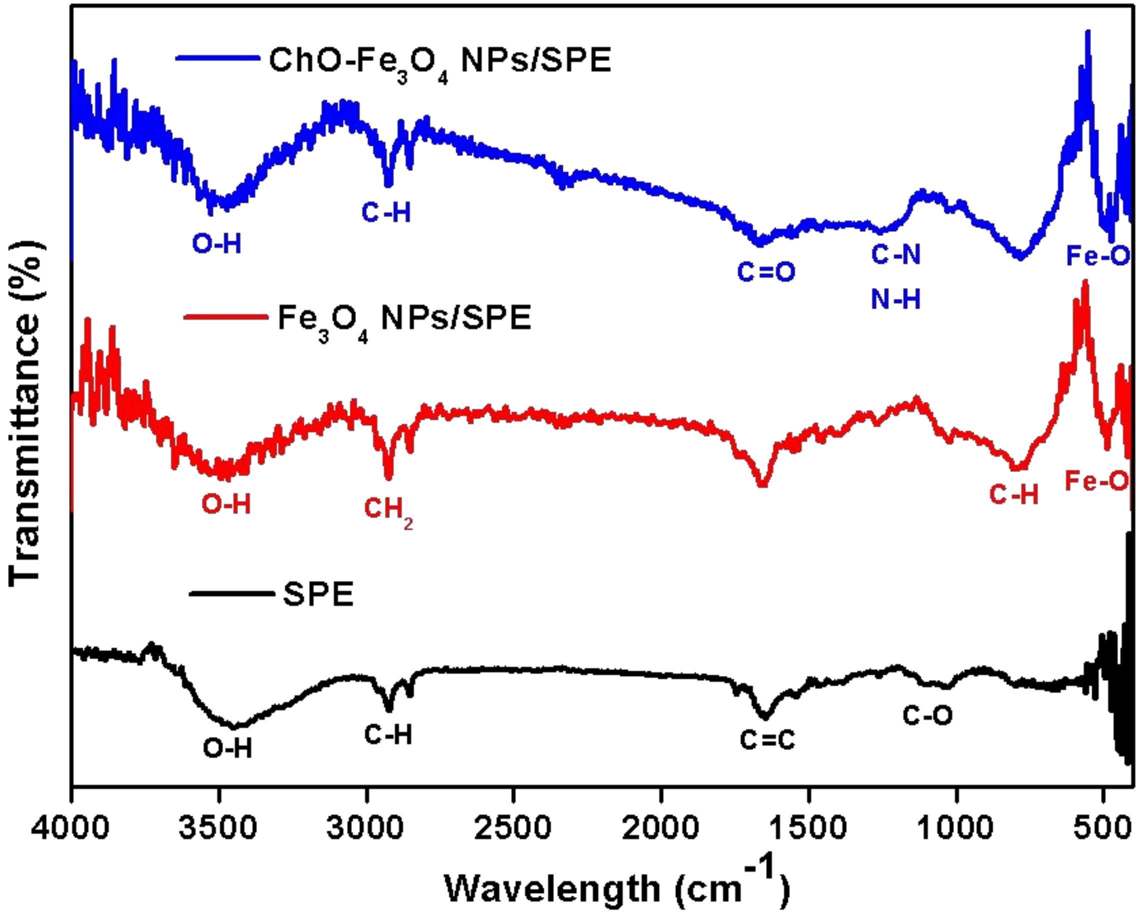

Figure S1. *Solid-state FTIR of SPE (black), Fe_3O_4 NPs/SPE (red), and ChO–Fe_3O_4 NPs/SPE (blue) for confirming physisorbed ChO on the Fe_3O_4 film.*

S5. XPS Analysis

Table S2. *Quantification Report of Fe_3O_4 NPs from XPS analysis presented in* ***Figure S2A-C****.*

Peak	Position binding energy (eV)	FWHM (eV)	Raw Area (cps eV)	RSF	Atomic Mass	Atomic Conc%	Mass Conc%
**O 1s**	527.00	3.868	212410	0.780	15.999	67.06	36.84
**Fe 2p**	708.00	4.534	371280	2.957	55.846	32.94	63.16

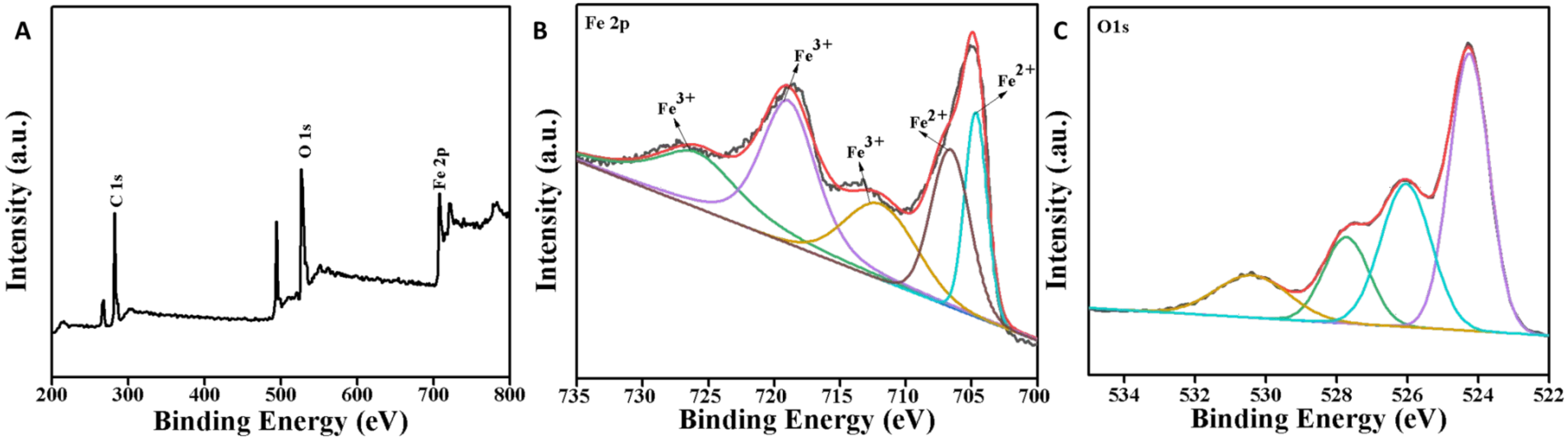

Figure S2. *XPS spectra of A) survey scan, B) Fe 2p, and C) O 1s for Fe_3O_4 NPs.*

S6. Details of Variables in Equations (Kinetics Studies)

This section provides the details of variables used in **Eq. 5 to 7**, for calculating K_s in **Eq. 5** the variables 'm' represents the peak-to-peak separation (0.28 V), 'v' is the scan rate (50 mV s^{-1}), 'n' denotes the number of electrons transferred in the redox process (n = 1), 'T' is the temperature (27 °C), 'R' is the gas constant (8.314 J mol^{-1} K^{-1}), and 'F' is the Faraday constant (96,485 C mol^{-1}). For **Eq. 6**, applied to calculate the D in a one-electron transfer and quasi-reversible electrochemical reaction system, 'α' is the charge transfer coefficient (0.879), calculated using **Eq. S1** with I_{pa} (0.10077 mA) and I_{pc} (-0.11463 mA) values at a 50 mV s^{-1} scan rate. 'C' represents the mediator concentration (0.000005 mol cm^{-3}), while other variables (R, F, T, n, ν) remain consistent with **Eq. 5**. Finally, in **Eq. 7**, used for determining Υ, 'A' denotes the electrode surface area (0.0707 cm^2), with other variables identical to those in **Eqs. 5 & 6**. These equations collectively highlight the electrochemical kinetics of the fabricated ChO-Fe_3O_4 NPs/SPE nanobioengineered electrode, underscoring its potential for effective malathion sensing.

$$\alpha = \frac{I_{pa}}{I_{pc}} \qquad \textbf{Eq. S1}$$

S7. Enzyme Loading, Reproducibility, Stability, and Real Sample Analysis

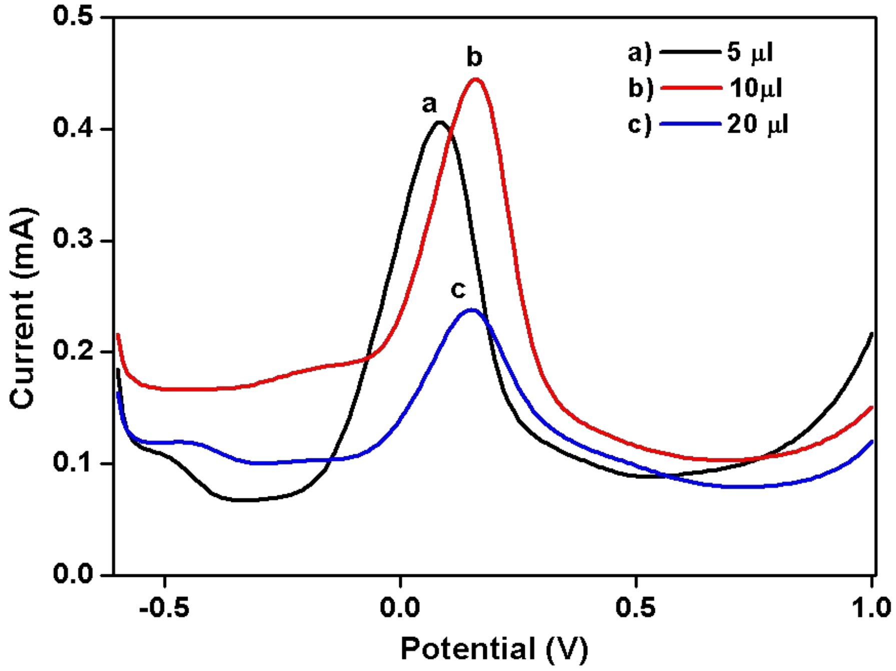

Figure S3. *DPV responses of ChO-Fe_3O_4 NPs/SPE electrodes prepared with varying ChO loadings (5 µL, 10 µL, and 20 µL). The 10 µL enzyme loading showed the most balanced current response and stability, indicating optimal catalytic efficiency for subsequent biosensing experiments.*

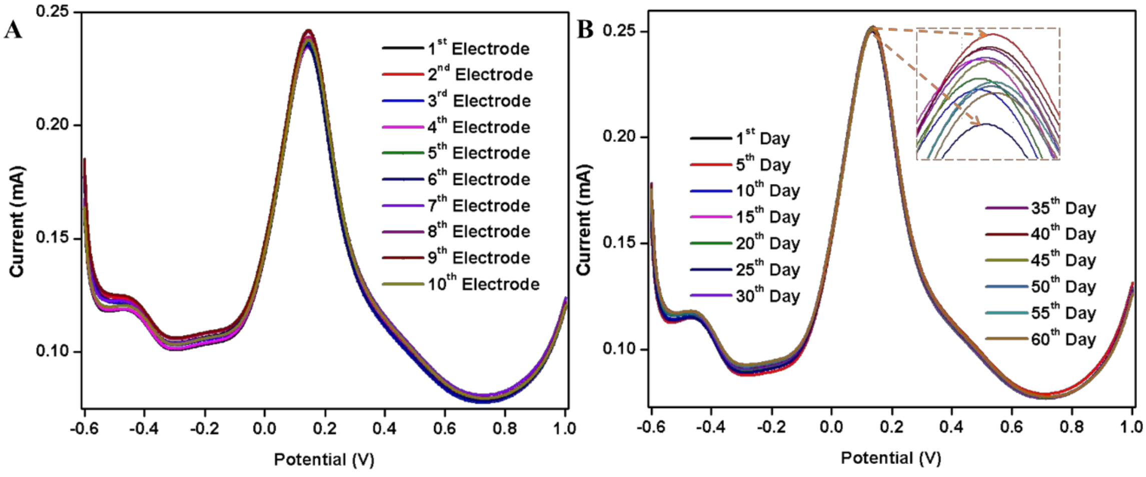

Figure S4. *DPV Scan: A) reproducibility and B) stability analysis.*

**Table S3.** *Malathion in spiked soil samples (Real sample analysis).*			
**Concentration of malathion added (µM)**	**Concentration of malathion determined in soil samples (µM)**	**Recovery (%)**	**RSD (%)**
10	9.61	96.17	0.77
60	56.37	93.95	0.11
100	98.42	98.42	0.87
140	136.08	97.20	0.009

A CH. 1 C.S 2.50 ATT 10 OFFS 0 00/00/00 00:03

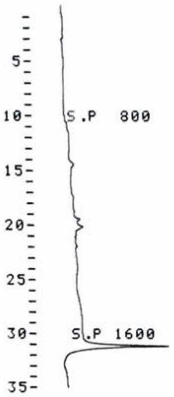

D-2500 00/00/00 00:03

METHOD: TAG: 15 CH: 1

FILE: 3 CALC-METHOD: AREA% TABLE: 0 CONC: AREA

NO.	RT	AREA	CONC	BC
3	4.98	225786	3.582	BB
4	11.71	414204	6.571	BV
5	12.72	295026	4.680	VV
6	14.43	325790	5.168	VB
7	17.50	116597	1.850	BV
8	18.76	146297	2.321	VV
9	19.46	256321	4.066	VV
10	20.15	473035	7.504	VV
13	21.95	116235	1.844	VV
14	23.87	346038	5.490	VB
15	31.18	3588195	56.924	BB
TOTAL				
		6303524	100.000	

PEAK REJ : 100000

B CH. 1 C.S 2.50 ATT 10 OFFS 0 00/00/00 00:42

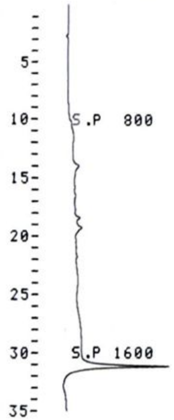

D-2500 00/00/00 00:42

METHOD: TAG: 16 CH: 1

FILE: 3 CALC-METHOD: AREA% TABLE: 0 CONC: AREA

NO.	RT	AREA	CONC	BC
1	11.40	325824	6.437	BV
2	11.80	100276	1.981	VV
4	13.98	280882	5.549	BB
8	18.47	162774	3.216	VV
9	19.31	353837	6.991	VV
13	23.99	245328	4.847	VB
14	31.18	3592687	70.979	BB
TOTAL				
		5061608	100.000	

PEAK REJ : 100000

Figure S5. *HPLC analysis: A) Blank run in Methanol and B) 200 µM Malathion.*
